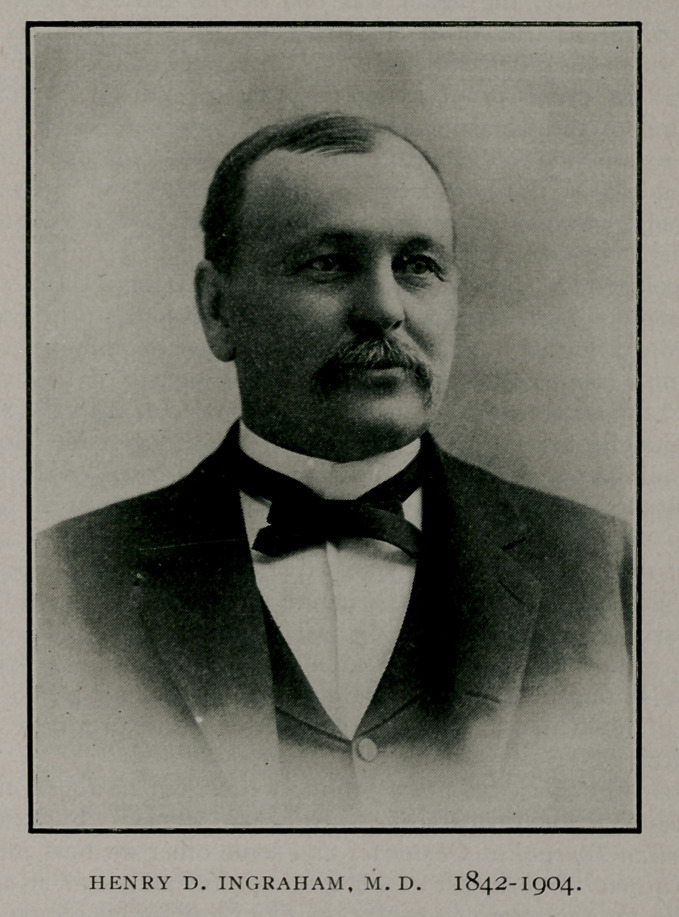# Dr. Henry D. Ingraham

**Published:** 1904-07

**Authors:** 


### OBITUARY.

Dr. Henry D. Ingraham died at his home in Buffalo, May 23, 1904, after a prolonged illness, aged 62 years. He was a native of New Hampshire, but his youth and early manhood were spent in the neighborhood of Arcade, N. Y., where he was educated at the common schools and at the Arcade Seminary. For a time

HENRY D. INGRAHAM, M. D. 1842-1904.

he taught in the common schools, and in 1863 began the study of medicine in the office of the late Dr. Lucius Peck, of Arcade. He attended medical lectures at the University of Buffalo, and graduated from that institution February 21, 1866.

Dr. Ingraham began the practice of medicine at East Randolph, N. Y., but soon settled at Kennedy, N. Y., where he associated himself with the late Dr. William Smith. He established there a large and lucrative practice, remaining until 1880, when he removed to Jamestown, N. Y., but after a few months decided to locate at Buffalo. He came here in 1881, and this city has since been the scene of his activities until his death. He developed a special liking for gynecological surgery and, while never entirely relinquishing family practice, he came to be recognised as one of the leading gynecologists, and in this department of practice he won the confidence and following of a large professional circle.

In 1883, Dr. Ingraham was one of the active organisers of the medical department of Niagara University, and until its union with the medical department of the University of Buffalo in 1898, he was its professor of gynecology and pediatrics. In the upbuilding of the department, he was an important factor. From 1898 to 1902, he was clinical professor of gynecology and diseases of children of the University of Buffalo. In 1883, he was appointed gynecologist to the Buffalo Hospital of the Sisters of Charity. Besides serving this institution most creditably as its gynecologist, he did much to advance its interests by supervising the erection of the new wing and other additions to the hospital, by coöperating with its authorities in many of the minor details of administration, and by aiding the establishment of the training school for nurses. After severing his connection with the Sisters' Hospital he became the gynecologist to the Riverside Hospital, continuing as such until his death. He was also one of the gynecologists to the Erie County Hospital from the time of its organisation until his death.

In medical societies he was active and influential. He was a member of the American Association of Obstetricians and Gynecologists, of the American Medical Association and also of the state, county and city organisations. In 1902, he was chairman of the section of gynecology and obstetrics of the Buffalo Academy of Medicine, and at the time of his death president of the Medical Union of Buffalo.

Dr. Ingraham was not a prolific writer, but he found time to publish occasional papers in the BUFFALO MEDICAL JOURNAL, the *American Journal of Obstetrics* and some other medical journals.

No man of character and ability can live and work in a community for a quarter of a century without leaving his impress upon it, this being especially true of the successful physician, and such was Dr. Ingraham.

He is survived by a wife and step-daughter, by three sisters, and by several nieces and nephews, among the latter of whom is Dr. Henry C. Buswell of this city. A. A. H.

## Figures and Tables

**Figure f1:**